# The chromosomal genome sequence of the bigfin reef squid, *Sepioteuthis lessoniana *d'Orbigny, 1826 and its associated microbial metagenome sequences

**DOI:** 10.12688/wellcomeopenres.24553.1

**Published:** 2025-07-14

**Authors:** Gustavo Sanchez, Oleg Simakov, Spencer Nyholm, Michele Nishiguchi, Margaret McFall-Ngai, Raphael Lami, Elizabeth Heath-Heckman, Graeme Oatley, Elizabeth Sinclair, Eerik Aunin, Noah Gettle, Camilla Santos, Michael Paulini, Haoyu Niu, Victoria McKenna, Rebecca O’Brien, Wellcome Sanger Institute Tree of Life Management, Samples and Laboratory Team, Wellcome Sanger Institute Scientific Operations: Sequencing Operations, Wellcome Sanger Institute Tree of Life Core Informatics Team, EBI Aquatic Symbiosis Genomics Data Portal Team, Aquatic Symbiosis Genomics Project Leadership

**Affiliations:** 1 Molecular Genetics Unit, Okinawa Institute of Science and Technology Graduate University, Onna, Okinawa, Japan; 2 Integrated Science for Life, Hiroshima University, Higashi Hiroshima, Hiroshima, Japan; 3 University of Vienna, Vienna, Vienna, Austria; 4 University of Connecticut, Storrs, Connecticut, USA; 5 University of California Merced, Merced, California, USA; 6 California Institute of Technology, Pasadena, California, USA; 7 Microbial Biodiversity et Biotechnology Lab, Sorbonne University, Banyuls-sur-mer, France; 8 Michigan State University, East Lansing, Michigan, USA; 9 Tree of Life, Wellcome Sanger Institute, Hinxton, England, UK

**Keywords:** Sepioteuthis lessoniana, bigfin reef squid, genome sequence, chromosomal, Myopsida

## Abstract

We present a genome assembly from a specimen of *Sepioteuthis lessoniana* (bigfin reef squid; Mollusca; Cephalopoda; Myopsida; Loliginidae). The genome sequence has a total length of 5,056.23 megabases. Most of the assembly (86.4%) is scaffolded into 44 chromosomal pseudomolecules. The mitochondrial genome has also been assembled and is 16.64 kilobases in length. Gene annotation of this assembly on Ensembl identified 28,970 protein-coding genes.

## Species taxonomy

Eukaryota; Opisthokonta; Metazoa; Eumetazoa; Bilateria; Protostomia; Spiralia; Lophotrochozoa; Mollusca; Cephalopoda; Coleoidea; Decapodiformes; Myopsida; Loliginidae; *Sepioteuthis*; *Sepioteuthis lessoniana* d'Orbigny, 1826 (NCBI:txid34570)

## Background

The bigfin reef squid *Sepioteuthis lessoniana* Férussac, 1831 in Lesson (1830–1831) is a demersal neritic species that belongs the Order Myopsida and the Family Loliginidae. *S. lessoniana* is a species complex with three lineages distributed across the coast of the Indo-Pacific Ocean, from the western Indian Ocean in the Red Sea to the Central Pacific Ocean (Hawaii) ( Cheng *et al.*, 2014). In Japanese waters, three cryptic species have been identified based on COI sequences, allozyme electrophoresis patterns and morphology, referred to as the “aka,” “shiro,” and “kua” types ( Imai & Aoki, 2012; Tomano *et al.*, 2016). These types correspond to *Sepioteuthis* sp. 1, sp. 2, and sp. 3, respectively, which represent the three distinct lineages found throughout the entire distribution of the *S. lessoniana* sensu stricto. The “aka” and “shiro” types are abundant off mainland Japanese waters, while the “kua,” “shiro,” and “aka” types are all found in the waters of the Ryukyu Archipelago. In addition, the “kua” type is absent from mainland Japanese waters, while the “shiro” type is the most dominant species in mainland Japan.

Hatchlings of *Sepioteuthis* sp. 2 are larger and more developed than any other loliginids. This advanced development has enabled successful culturing of this species over several generations ( Arnold, 1990; Shigeno *et al.*, 2001), the study of social behaviour such as schooling ( Sakurai & Ikeda, 2024; Sugimoto *et al.*, 2013; Sugimoto & Ikeda, 2012) and camouflage ( Nakajima *et al.*, 2022). A reference genome for this species will facilitate comparative genomics in the Myopsida Order, enable research into the neural mechanisms underlying social behaviour, and support selective breeding through genotyping, ultimately advancing aquaculture practices.

## Genome sequence report

### Sequencing data

**Figure 1.**
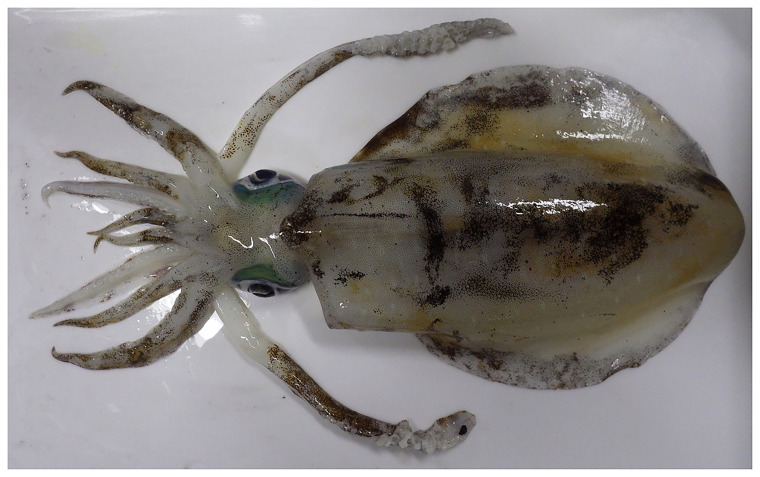
Photograph of the *Sepioteuthis lessoniana* (xcSepLess1) specimen used for genome sequencing. (Photo Credits to Dr Satoshi Tomano.)

The genome of a specimen of *Sepioteuthis lessoniana* ( Figure 1) was sequenced using Pacific Biosciences single-molecule HiFi long reads, generating 249.90 Gb from 30.19 million reads. Based on the estimated genome size, the sequencing data provided approximately 34× coverage of the genome. Chromosome conformation Hi-C data produced 1,203.59 Gb from 7,970.82 million reads. RNA sequencing data were also generated and are available in public sequence repositories. Table 1 summarises the specimen and sequencing information.

**Table 1.** Specimen and sequencing data for *Sepioteuthis lessoniana*.

Project information			
**Study title**	Sepioteuthis lessoniana (bigfin reef squid)		
**Umbrella BioProject**	PRJEB64979		
**Species**	*Sepioteuthis lessoniana*		
**BioSpecimen**	SAMEA11646235		
**NCBI taxonomy ID**	34570		
Specimen information			
**Technology**	**ToLID**	**BioSample accession**	**Organism part**
**PacBio long read sequencing**	xcSepLess1	SAMEA11646288	Somatic tissue
**Hi-C sequencing**	xcSepLess2	SAMEA11646318	Somatic tissue
**RNA sequencing**	xcSepLess2	SAMEA11646297	Gill
Sequencing information			
**Platform**	**Run accession**	**Read count**	**Base count (Gb)**
**Hi-C Illumina NovaSeq 6000**	ERR11837536	3.61e+09	545.23
**Hi-C Illumina NovaSeq 6000**	ERR11837535	4.36e+09	658.36
**PacBio Sequel IIe**	ERR11843445	2.75e+06	19.73
**PacBio Sequel IIe**	ERR11843447	2.69e+06	22.08
**PacBio Sequel IIe**	ERR11843448	2.63e+06	25.16
**PacBio Sequel IIe**	ERR11843449	2.64e+06	23.25
**PacBio Sequel IIe**	ERR11843451	2.88e+06	19.13
**PacBio Revio**	ERR12055552	8.23e+06	69.17
**PacBio Sequel IIe**	ERR11843446	2.94e+06	20.88
**PacBio Sequel IIe**	ERR11843450	2.93e+06	21.37
**PacBio Sequel IIe**	ERR11843452	2.49e+06	29.14
**RNA Illumina NovaSeq X**	ERR13093641	1.36e+08	20.58
**RNA Illumina NovaSeq 6000**	ERR11837537	4.42e+07	6.68

### Assembly statistics

The primary haplotype was assembled, and contigs corresponding to an alternate haplotype were also deposited in INSDC databases. The assembly was improved by manual curation, which corrected 577 misjoins or missing joins and removed 3 haplotypic duplications. These interventions reduced the total assembly length by 0.35%, decreased the scaffold count by 4.09%, and increased the scaffold N50 by 14.75%. The final assembly has a total length of 5,056.23 Mb in 6,819 scaffolds, with 7,097 gaps, and a scaffold N50 of 96.87 Mb ( Table 2).

**Table 2.** Genome assembly data for *Sepioteuthis lessoniana*.

Genome assembly		
Assembly name	xcSepLess1.1	
Assembly accession	GCA_963585895.1	
*Alternate haplotype accession*	*GCA_963585875.1*	
Assembly level for primary assembly	chromosome	
Span (Mb)	5,056.23	
Number of contigs	13,916	
Number of scaffolds	6,819	
Longest scaffold (Mb)	165.03	
Assembly metric	Measure	*Benchmark*
Contig N50 length	0.66 Mb	*≥ 1 Mb*
Scaffold N50 length	96.87 Mb	*= chromosome N50*
Consensus quality (QV)	Primary: 51.5; alternate: 52.7; combined 52.0	*≥ 40*
*k*-mer completeness	Primary: 84.36%; alternate: 81.77%; combined: 94.54%	*≥ 95%*
BUSCO *	C:73.7%[S:72.7%,D:1.0%],F:4.8%,M:21.4%,n:5,295	*S > 90%* *D < 5%*
Percentage of assembly assigned to chromosomes	86.38%	*≥ 90%*
Organelles	Mitochondrial genome: 16.64 kb	*complete single alleles*
Genome annotation of assembly GCA_963585895.1 at Ensembl		
Number of protein-coding genes	28,970	
Number of non-coding genes	21,583	
Number of gene transcripts	68,971	

* BUSCO scores based on the mollusca_odb10 BUSCO set using version 5.5.0. C = complete [S = single copy, D = duplicated], F = fragmented, M = missing, n = number of orthologues in comparison.

**Figure 2.**
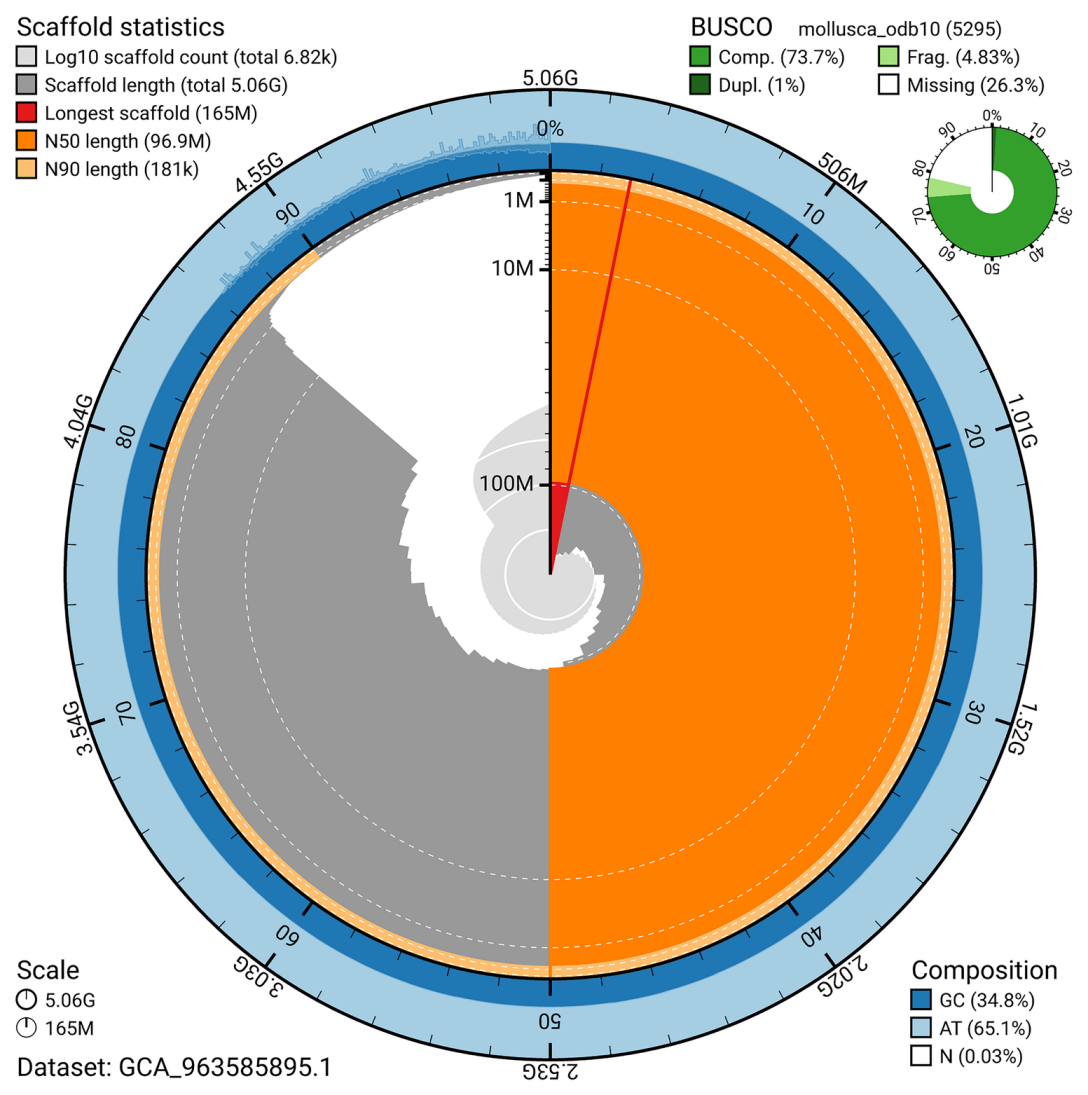
Genome assembly of *Sepioteuthis lessoniana*, xcSepLess1.1: metrics. The BlobToolKit snail plot provides an overview of assembly metrics and BUSCO gene completeness. The circumference represents the length of the whole genome sequence, and the main plot is divided into 1,000 bins around the circumference. The outermost blue tracks display the distribution of GC, AT, and N percentages across the bins. Scaffolds are arranged clockwise from longest to shortest and are depicted in dark grey. The longest scaffold is indicated by the red arc, and the deeper orange and pale orange arcs represent the N50 and N90 lengths. A light grey spiral at the centre shows the cumulative scaffold count on a logarithmic scale. A summary of complete, fragmented, duplicated, and missing BUSCO genes in the mollusca_odb10 set is presented at the top right. An interactive version of this figure is available at https://blobtoolkit.genomehubs.org/view/GCA_963585895.1/dataset/GCA_963585895.1/snail.

**Figure 3.**
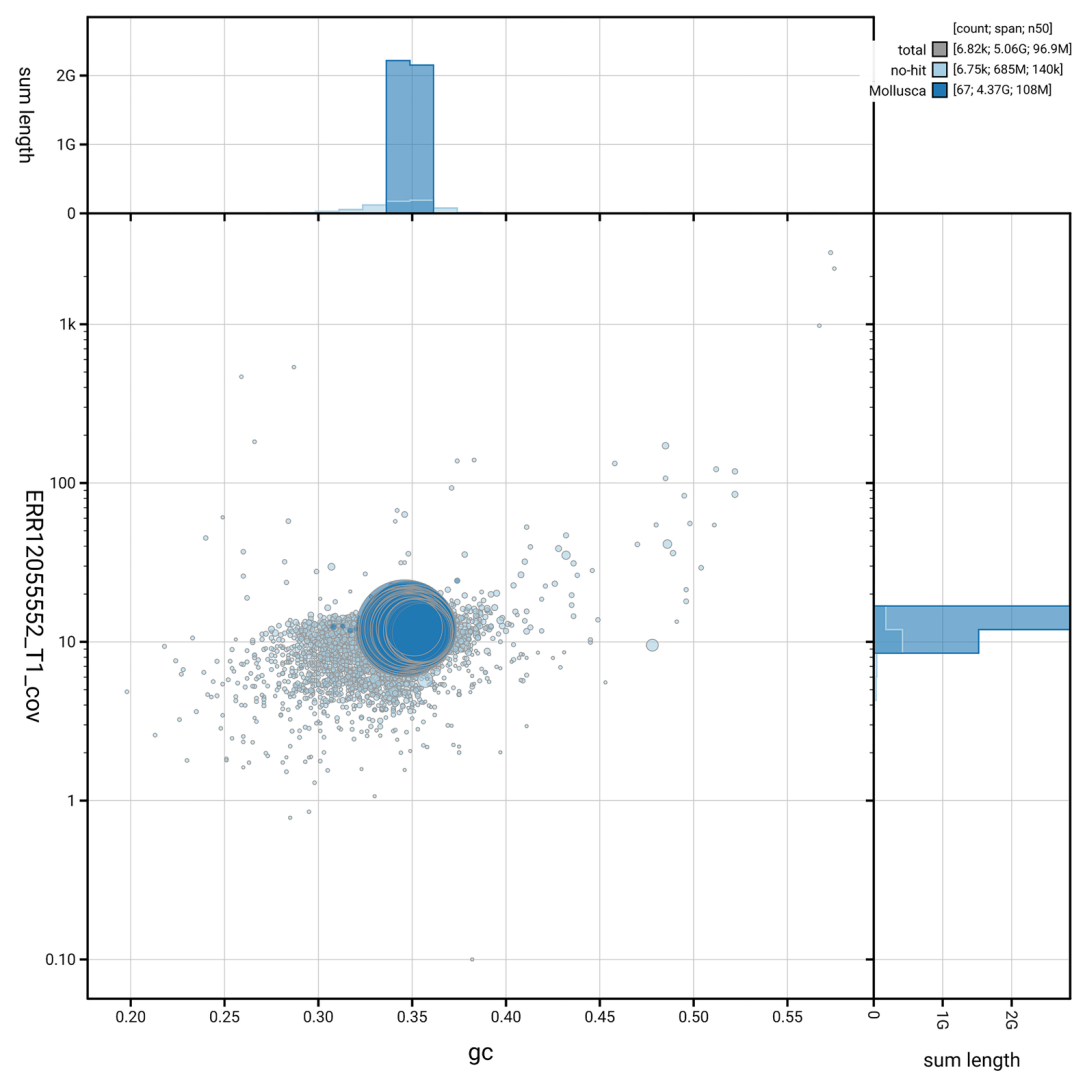
Genome assembly of *Sepioteuthis lessoniana*, xcSepLess1.1: BlobToolKit GC-coverage plot. Blob plot showing sequence coverage (vertical axis) and GC content (horizontal axis). The circles represent scaffolds, with the size proportional to scaffold length and the colour representing phylum membership. The histograms along the axes display the total length of sequences distributed across different levels of coverage and GC content. An interactive version of this figure is available at https://blobtoolkit.genomehubs.org/view/GCA_963585895.1/dataset/GCA_963585895.1/blob.

**Figure 4.**
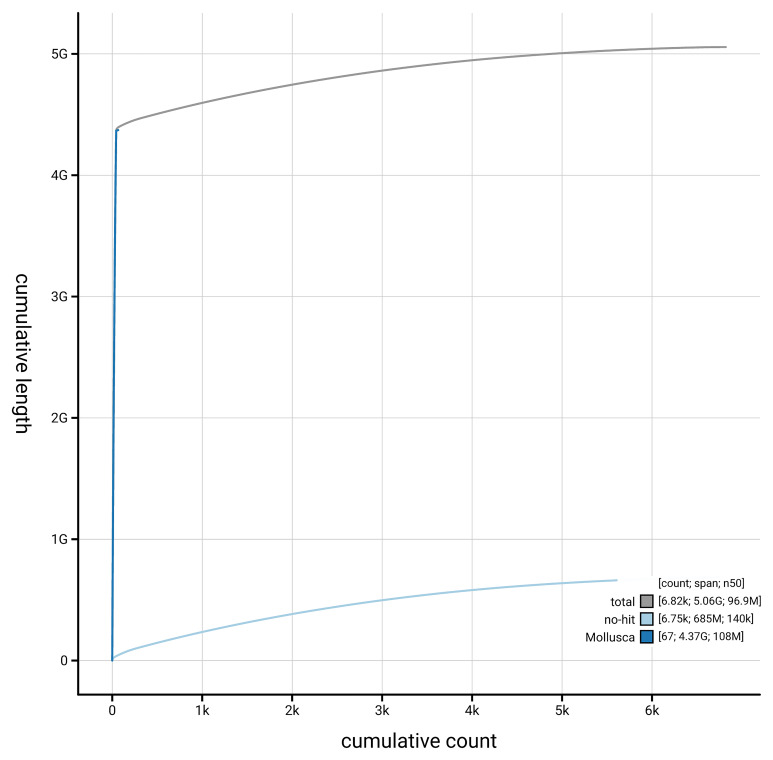
Genome assembly of *Sepioteuthis lessoniana,* xcSepLess1.1: BlobToolKit cumulative sequence plot. The grey line shows cumulative length for all scaffolds. Coloured lines show cumulative lengths of scaffolds assigned to each phylum using the buscogenes taxrule. An interactive version of this figure is available at https://blobtoolkit.genomehubs.org/view/GCA_963585895.1/dataset/GCA_963585895.1/cumulative.

The snail plot in Figure 2 provides a summary of the assembly statistics, indicating the distribution of scaffold lengths and other assembly metrics. Figure 3 shows the distribution of scaffolds by GC proportion and coverage. Figure 4 presents a cumulative assembly plot, with separate curves representing different scaffold subsets assigned to various phyla, illustrating the completeness of the assembly.

**Figure 5.**
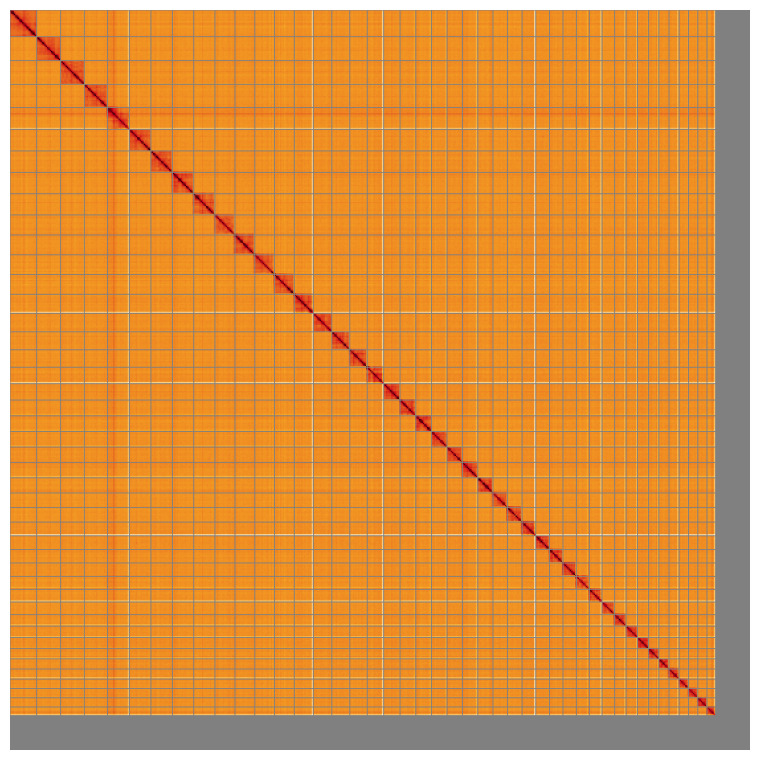
Genome assembly of *Sepioteuthis lessoniana*, xcSepLess1.1: Hi-C contact map of the xcSepLess1.1 assembly, visualised using HiGlass. Chromosomes are shown in order of size from left to right and top to bottom. An interactive version of this figure may be viewed at https://genome-note-higlass.tol.sanger.ac.uk/l/?d=RfIiARSCSauoz1ZtvLxtng.

Most of the assembly sequence (86.38%) was assigned to 44 chromosomal-level scaffolds. These chromosome-level scaffolds, confirmed by Hi-C data, are named according to size ( Figure 5; Table 3). During curation, it was noted that a large amount of repeat sequences could not be placed on chromosomes.

**Table 3.** Chromosomal pseudomolecules in the genome assembly of *Sepioteuthis lessoniana*, xcSepLess1.

INSDC accession	Name	Length (Mb)	GC%
OY757904.1	1	165.03	34.5
OY757905.1	2	148.29	34.5
OY757906.1	3	146.83	34.5
OY757907.1	4	143.61	34.5
OY757908.1	5	135.24	35
OY757909.1	6	133.26	35
OY757910.1	7	133.09	35
OY757911.1	8	131.98	34.5
OY757912.1	9	131.16	34.5
OY757913.1	10	124.05	34.5
OY757914.1	11	122.65	35
OY757915.1	12	122.42	35
OY757916.1	13	122.24	34.5
OY757917.1	14	117.8	35
OY757918.1	15	114.22	35
OY757919.1	16	111.07	35
OY757920.1	17	108.32	34.5
OY757921.1	18	101.85	35
OY757922.1	19	101.23	35.5
OY757923.1	20	97.57	35
OY757924.1	21	96.87	35
OY757925.1	22	96.03	35
OY757926.1	23	95.44	35.5
OY757927.1	24	95.42	35
OY757928.1	25	93.84	35
OY757929.1	26	90.61	34.5
OY757930.1	27	89.63	35
OY757931.1	28	84.68	35.5
OY757932.1	29	84.74	35.5
OY757933.1	30	83.72	35
OY757934.1	31	82.93	35
OY757935.1	32	80.29	35
OY757936.1	33	78.34	35
OY757937.1	34	78.08	35
OY757938.1	35	71.07	35
OY757939.1	36	70.69	35.5
OY757940.1	37	68.51	35.5
OY757941.1	38	63.29	35.5
OY757942.1	39	62.91	35.5
OY757943.1	40	62.5	35.5
OY757944.1	41	58.21	35.5
OY757945.1	42	57.4	35
OY757946.1	43	57.12	35.5
OY757947.1	44	53.38	35
OY757948.1	MT	0.02	29

The mitochondrial genome was also assembled. This sequence is included as a contig in the multifasta file of the genome submission and as a standalone record in GenBank.

### Assembly quality metrics

The estimated Quality Value (QV) and *k*-mer completeness metrics, along with BUSCO completeness scores, were calculated for each haplotype and the combined assembly. The QV reflects the base-level accuracy of the assembly, while *k*-mer completeness indicates the proportion of expected *k*-mers identified in the assembly. BUSCO scores provide a measure of completeness based on benchmarking universal single-copy orthologues.

The combined primary and alternate assemblies achieve an estimated QV of 52.0. The *k*-mer completeness is 84.36% for the primary haplotype and 81.77% for the alternate haplotype; and 94.54% for the combined primary and alternate assemblies. BUSCO v.5.5.0 analysis using the mollusca_odb10 reference set ( *n* = 5,295) identified 73.7% of the expected gene set (single = 72.7%, duplicated = 1.0%).

## Metagenome report

**Figure 6.**
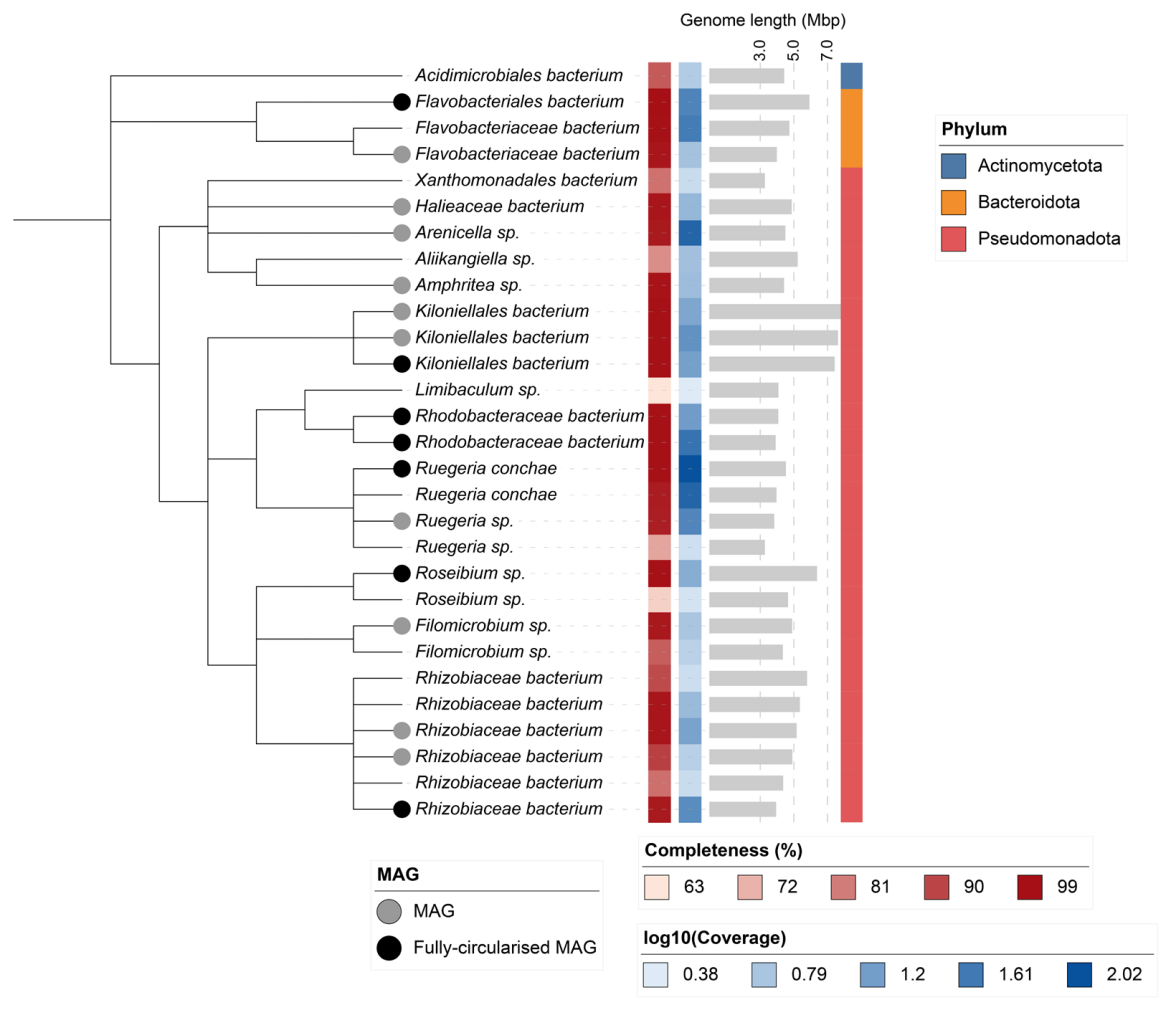
Cladogram showing the taxonomic placement of metagenome bins, constructed using NCBI taxonomic identifiers with *taxonomizr* and annotated in iTOL. Colours indicate phylum-level taxonomy. Additional tracks show sequencing coverage (log₁₀), genome size (Mbp), and completeness. Bins that meet the criteria for MAGs are marked with a grey circle; fully circularised MAGs are marked in black.

Twenty-nine binned genomes were generated from the metagenome assembly, of which 17 were classified as high-quality metagenome assembled genomes (MAGs) (see methods). The completeness values for these assemblies range from approximately 63% to 100% with contamination below 9%. A cladogram of the binned metagenomes is shown in Figure 6. For details on binned genomes see Table 4.

**Table 4.** Quality metrics and taxonomic assignments of the binned metagenomes.

NCBI taxon	Taxid	GTDB taxonomy	Quality	Size (bp)	Contigs	Circular	Mean coverage	Completeness (%)	Contamination (%)
Ruegeria sp.	259304	g__Ruegeria	High	3,805,421	1	No	29.78	97.34	0.53
Rhodobacteraceae bacterium	157276	g__SZUA-547	High	3,886,458	1	Yes	46.65	99.6	0.6
Rhizobiaceae bacterium	271151	f__Rhizobiaceae	High	3,917,026	1	Yes	25.66	97.91	1.23
Ruegeria conchae	981384	s__Ruegeria conchae	High	3,942,089	1	Yes	66.83	97.66	0.32
Flavobacteriaceae bacterium	165436	g__SMXJ01	High	3,961,667	3	No	6.69	98.76	0.41
Rhodobacteraceae bacterium	157276	g__SZUA-547	High	4,052,132	1	Yes	16.84	99.6	0.6
Amphritea sp.	981605	g__Amphritea	High	4,385,521	2	No	7.67	99.29	1
Arenicella sp.	1586337	g__Arenicella	High	4,474,314	19	Partial	65.39	98.17	0.61
Ruegeria conchae	981384	s__Ruegeria conchae	High	4,502,159	2	Yes	104.23	99.49	0.43
Halieaceae bacterium	2735679	f__Halieaceae	High	4,838,237	1	No	8.96	98.7	1.99
Filomicrobium sp.	293328	g__Filomicrobium	High	4,869,283	35	Partial	6.22	98.39	2.35
Rhizobiaceae bacterium	271151	f__Rhizobiaceae	High	4,883,131	32	No	4.87	91.73	2.09
Rhizobiaceae bacterium	271151	f__Rhizobiaceae	High	5,142,339	7	No	14.59	98.8	4.78
Flavobacteriales bacterium	213322	o__Flavobacteriales	High	5,895,559	1	Yes	29.94	99.73	1.34
Roseibium sp.	1936171	g__Roseibium	High	6,353,716	1	Yes	10.96	99.58	0.63
Kiloniellales bacterium	1667039	g__JACOMY01	High	7,402,078	1	Yes	15.46	99.57	0.87
Kiloniellales bacterium	1667039	g__JACOMY01	High	7,596,607	1	No	21.94	99.57	0.43
Kiloniellales bacterium	1667039	g__JACOMY01	High	7,789,244	1	No	13.27	99.57	0.22
Xanthomonadales bacterium	412058	f__SZUA-36	Medium	3,248,922	30	No	3.55	83.14	0.91
Ruegeria sp.	259304	s__Ruegeria sp003443535	Medium	3,249,342	27	No	3.29	74.63	0
Limibaculum sp.	2036014	g__Limibaculum	Medium	4,064,720	170	No	2.4	63.37	1.2
Filomicrobium sp.	293328	g__Filomicrobium	Medium	4,321,939	42	No	4.53	86.38	1.81
Rhizobiaceae bacterium	271151	g__JAALLB01	Medium	4,339,548	49	No	3.58	83.5	1.34
Acidimicrobiales bacterium	310071	f__Bin134	Medium	4,404,047	14	No	5.15	87.18	2.99
Roseibium sp.	1936171	g__Roseibium	Medium	4,625,544	74	No	2.97	67.24	3.45
Flavobacteriaceae bacterium	165436	g__GCA-2733415	Medium	4,710,532	7	No	36.9	99.53	7.38
Aliikangiella sp.	1920244	g__Aliikangiella	Medium	5,204,448	13	No	7.08	78.38	0.27
Rhizobiaceae bacterium	271151	f__Rhizobiaceae	Medium	5,326,527	32	No	8.06	98.8	5.26
Rhizobiaceae bacterium	271151	g__SPNT01	Medium	5,757,256	129	Partial	3.41	89.74	8.83

## Genome annotation report

The *Sepioteuthis lessoniana* genome assembly (GCA_963585895.1) was annotated at the European Bioinformatics Institute (EBI) on Ensembl Rapid Release. The resulting annotation includes 68,971 transcribed mRNAs from 28,970 protein-coding and 21,583 non-coding genes ( Table 2; https://beta.ensembl.org/species/f1e0d42d-d087-4e33-b0af-a3606c35ba3b). The average transcript length is 27,933.39, with 1.36 coding transcripts per gene and 4.95 exons per transcript.

## Methods

### Sample acquisition

The specimen used for genome sequencing, one female of *Sepioteuthis* sp. 2, the “shiro” type (specimen ID VIEC0000008, ToLID xcSepLess1), was collected using jigging off Tosa Bay in Kochi Prefecture on 2021-04-23. The specimen was shipped frozen at –20 °C to Hiroshima University. The muscle mantle of this specimen was preserved in a 2mL Eppendorf tube and kept at –20 °C.

The specimen used for Hi-C and RNA sequencing (specimen ID VIEC0000009, ToLID xcSepLess2) was an adult specimen collected from Shimane Prefecturen, Hamada, Japan on 2021-05-21 by jigging. The specimens were collected and identified by Gustavo Sanchez (Hiroshima University).

### Nucleic acid extraction

The workflow for high molecular weight (HMW) DNA extraction at the Wellcome Sanger Institute (WSI) Tree of Life Core Laboratory includes a sequence of procedures: sample preparation and homogenisation, DNA extraction, fragmentation and purification. Detailed protocols are available on protocols.io ( Denton *et al.*, 2023). The xcSepLess1 sample was prepared for DNA extraction by weighing and dissecting it on dry ice ( Jay *et al.*, 2023). Tissue from the muscle tissue was cryogenically disrupted using the Covaris cryoPREP ^®^ Automated Dry Pulverizer ( Narváez-Gómez *et al.*, 2023). HMW DNA was extracted using the Automated MagAttract v2 protocol ( Oatley *et al.*, 2023). DNA was sheared into an average fragment size of 12–20 kb in a Megaruptor 3 system ( Bates *et al.*, 2023). Sheared DNA was purified by solid-phase reversible immobilisation, using AMPure PB beads to eliminate shorter fragments and concentrate the DNA ( Strickland *et al.*, 2023). The concentration of the sheared and purified DNA was assessed using a Nanodrop spectrophotometer and Qubit Fluorometer using the Qubit dsDNA High Sensitivity Assay kit. Fragment size distribution was evaluated by running the sample on the FemtoPulse system.

RNA was extracted from gill tissue of xcSepLess2 in the Tree of Life Laboratory at the WSI using the RNA Extraction: Automated MagMax™ *mir*Vana protocol ( do Amaral *et al.*, 2023). The RNA concentration was assessed using a Nanodrop spectrophotometer and a Qubit Fluorometer using the Qubit RNA Broad-Range Assay kit. Analysis of the integrity of the RNA was done using the Agilent RNA 6000 Pico Kit and Eukaryotic Total RNA assay.

### Sequencing

Pacific Biosciences HiFi circular consensus DNA sequencing libraries were constructed according to the manufacturers’ instructions. DNA sequencing was performed by the Scientific Operations core at the WSI on Pacific Biosciences Sequel IIe. Tissue from the somatic tissue of the xcSepLess2 sample was processed for Hi-C sequencing at the WSI Scientific Operations core, using the Arima-HiC v2 kit.and sequenced on the Illumina NovaSeq 6000 instrument. Poly(A) RNA-Seq libraries were constructed using the NEB Ultra II RNA Library Prep kit, following the manufacturer’s instructions. RNA sequencing was performed on the Illumina NovaSeq 6000 instrument.

### Genome assembly, curation and evaluation

***Assembly***

Prior to assembly of the PacBio HiFi reads, a database of *k*-mer counts ( *k* = 31) was generated from the filtered reads using FastK. GenomeScope2 ( Ranallo-Benavidez *et al.*, 2020) was used to analyse the *k*-mer frequency distributions, providing estimates of genome size, heterozygosity, and repeat content.

The HiFi reads were assembled using Hifiasm ( Cheng *et al.*, 2021) with the --primary option. Haplotypic duplications were identified and removed using purge_dups ( Guan *et al.*, 2020). The Hi-C reads were mapped to the primary contigs using bwa-mem2 ( Vasimuddin *et al.*, 2019). The contigs were further scaffolded using the provided Hi-C data ( Rao *et al.*, 2014) in YaHS ( Zhou *et al.*, 2023) using the --break option for handling potential misassemblies. The scaffolded assemblies were evaluated using Gfastats ( Formenti *et al.*, 2022), BUSCO ( Manni *et al.*, 2021) and MERQURY.FK ( Rhie *et al.*, 2020).

The mitochondrial genome was assembled using MitoHiFi ( Uliano-Silva *et al.*, 2023), which runs MitoFinder ( Allio *et al.*, 2020) and uses these annotations to select the final mitochondrial contig and to ensure the general quality of the sequence.

***Assembly curation***

The assembly was decontaminated using the Assembly Screen for Cobionts and Contaminants (ASCC) pipeline. Flat files and maps used in curation were generated via the TreeVal pipeline ( Pointon *et al.*, 2023). Manual curation was conducted primarily in PretextView ( Harry, 2022) and HiGlass ( Kerpedjiev *et al.*, 2018), with additional insights provided by JBrowse2 ( Diesh *et al.*, 2023). Scaffolds were visually inspected and corrected as described by Howe *et al*. (2021). Any identified contamination, missed joins, and mis-joins were amended, and duplicate sequences were tagged and removed. The curation process is documented at https://gitlab.com/wtsi-grit/rapid-curation.

***Assembly quality assessment***

The Merqury.FK tool ( Rhie *et al.*, 2020), run in a Singularity container ( Kurtzer *et al.*, 2017), was used to evaluate *k*-mer completeness and assembly quality for the primary and alternate haplotypes using the *k*-mer databases ( *k* = 31) that were computed prior to genome assembly. The analysis outputs included assembly QV scores and completeness statistics.

A Hi-C contact map was produced for the final version of the assembly. The Hi-C reads were aligned using bwa-mem2 ( Vasimuddin *et al.*, 2019) and the alignment files were combined using SAMtools ( Danecek *et al.*, 2021). The Hi-C alignments were converted into a contact map using BEDTools ( Quinlan & Hall, 2010) and the Cooler tool suite ( Abdennur & Mirny, 2020). The contact map is visualised in HiGlass ( Kerpedjiev *et al.*, 2018).

The blobtoolkit pipeline is a Nextflow port of the previous Snakemake Blobtoolkit pipeline ( Challis *et al.*, 2020). It aligns the PacBio reads in SAMtools and minimap2 ( Li, 2018) and generates coverage tracks for regions of fixed size. In parallel, it queries the GoaT database ( Challis *et al.*, 2023) to identify all matching BUSCO lineages to run BUSCO ( Manni *et al.*, 2021). For the three domain-level BUSCO lineages, the pipeline aligns the BUSCO genes to the UniProt Reference Proteomes database ( Bateman *et al.*, 2023) with DIAMOND blastp ( Buchfink *et al.*, 2021). The genome is also divided into chunks according to the density of the BUSCO genes from the closest taxonomic lineage, and each chunk is aligned to the UniProt Reference Proteomes database using DIAMOND blastx. Genome sequences without a hit are chunked using seqtk and aligned to the NT database with blastn ( Altschul *et al.*, 1990). The blobtools suite combines all these outputs into a blobdir for visualisation.

The blobtoolkit pipeline was developed using nf-core tooling ( Ewels *et al.*, 2020) and MultiQC ( Ewels *et al.*, 2016), relying on the Conda package manager, the Bioconda initiative ( Grüning *et al.*, 2018), the Biocontainers infrastructure ( da Veiga Leprevost *et al.*, 2017), as well as the Docker ( Merkel, 2014) and Singularity ( Kurtzer *et al.*, 2017) containerisation solutions.

Table 5 contains a list of relevant software tool versions and sources.

**Table 5.** Software tools: versions and sources.

Software tool	Version	Source
BEDTools	2.30.0	https://github.com/arq5x/bedtools2
bin3C	0.3.3	https://github.com/cerebis/bin3C
BLAST	2.14.0	ftp://ftp.ncbi.nlm.nih.gov/blast/executables/blast+/
BlobToolKit	4.3.9	https://github.com/blobtoolkit/blobtoolkit
BUSCO	5.5.0	https://gitlab.com/ezlab/busco
bwa-mem2	2.2.1	https://github.com/bwa-mem2/bwa-mem2
CheckM	1.2.1	https://github.com/Ecogenomics/CheckM
Cooler	0.8.11	https://github.com/open2c/cooler
DIAMOND	2.1.8	https://github.com/bbuchfink/diamond
dRep	3.4.0	https://github.com/MrOlm/drep
fasta_windows	0.2.4	https://github.com/tolkit/fasta_windows
FastK	427104ea91c78c3b8b8b49f1a7d6bbeaa869ba1c	https://github.com/thegenemyers/FASTK
Gfastats	1.3.6	https://github.com/vgl-hub/gfastats
GoaT CLI	0.2.5	https://github.com/genomehubs/goat-cli
GTDB-TK	2.3.2	https://github.com/Ecogenomics/GTDBTk
Hifiasm	0.19.5-r587	https://github.com/chhylp123/hifiasm
HiGlass	44086069ee7d4d3f6f3f0012569789ec138f42b84aa44357826c0b6753eb28de	https://github.com/higlass/higlass
MaxBin	2.7	https://sourceforge.net/projects/maxbin/
MerquryFK	d00d98157618f4e8d1a9190026b19b471055b22e	https://github.com/thegenemyers/MERQURY.FK
MetaBat2	2.15-15-gd6ea400	https://bitbucket.org/berkeleylab/metabat/src/master/
MetaTOR	-	https://github.com/koszullab/metaTOR
Minimap2	2.24-r1122	https://github.com/lh3/minimap2
MitoHiFi	2	https://github.com/marcelauliano/MitoHiFi
MultiQC	1.14, 1.17, and 1.18	https://github.com/MultiQC/MultiQC
Nextflow	23.04.1	https://github.com/nextflow-io/nextflow
PretextView	0.2.5	https://github.com/sanger-tol/PretextView
PROKKA	1.14.5	https://github.com/vdejager/prokka
purge_dups	1.2.3	https://github.com/dfguan/purge_dups
samtools	1.19.2	https://github.com/samtools/samtools
sanger-tol/ascc	-	https://github.com/sanger-tol/ascc
sanger-tol/blobtoolkit	0.4.0	https://github.com/sanger-tol/blobtoolkit
Seqtk	1.3	https://github.com/lh3/seqtk
Singularity	3.9.0	https://github.com/sylabs/singularity
TreeVal	1.2.0	https://github.com/sanger-tol/treeval
YaHS	1.1a.2	https://github.com/c-zhou/yahs

### Genome annotation

The Ensembl Genebuild annotation system ( Aken *et al.*, 2016) was used to generate annotation for the *Sepioteuthis lessoniana* assembly (GCA_963585895.1) in Ensembl Rapid Release at the EBI. Annotation was created primarily through alignment of transcriptomic data to the genome, with gap filling via protein-to-genome alignments of a select set of proteins from UniProt ( UniProt Consortium, 2019).

### Metagenome assembly

The metagenome assembly was generated using MetaMDBG ( Benoit *et al.*, 2024) and binned using MetaBAT2 ( Kang *et al.*, 2019), MaxBin ( Wu *et al.*, 2014), bin3C ( DeMaere & Darling, 2019), and MetaTOR. The resulting bin sets of each binning algorithm were optimised and refined using MAGScoT ( Rühlemann *et al.*, 2022). PROKKA ( Seemann, 2014) was used to identify tRNAs and rRNAs in each bin, CheckM ( Parks *et al.*, 2015) (checkM_DB release 2015-01-16) was used to assess bin completeness/contamination, and GTDB-TK ( Chaumeil *et al.*, 2022) (GTDB release 214) was used to taxonomically classify bins. Taxonomic replicate bins were identified using dRep ( Olm *et al.*, 2017) with default settings (95% ANI threshold). The final bin set was filtered for bacteria and archaea. All bins were assessed for quality and categorised as metagenome-assembled genomes (MAGs) if they met the following criteria: contamination ≤ 5%, presence of 5S, 16S, and 23S rRNA genes, at least 18 unique tRNAs, and either ≥ 90% completeness or ≥ 50% completeness with fully circularised chromosomes. Bins that did not meet these thresholds, or were identified as taxonomic replicates of MAGs, were retained as ‘binned metagenomes’ provided they had ≥ 50% completeness and ≤ 10% contamination. A cladogram based on NCBI taxonomic assignments was generated using the ‘taxonomizr’ package in R. The tree was visualised and annotated using iTOL ( Letunic & Bork, 2024). Software tool versions and sources are given in Table 5.

### Wellcome Sanger Institute – Legal and Governance

The materials that have contributed to this genome note have been supplied by a Tree of Life collaborator. The Wellcome Sanger Institute employs a process whereby due diligence is carried out proportionate to the nature of the materials themselves, and the circumstances under which they have been/are to be collected and provided for use. The purpose of this is to address and mitigate any potential legal and/or ethical implications of receipt and use of the materials as part of the research project, and to ensure that in doing so we align with best practice wherever possible. The overarching areas of consideration are:

•   Ethical review of provenance and sourcing of the material

•   Legality of collection, transfer and use (national and international)

Each transfer of samples is undertaken according to a Research Collaboration Agreement or Material Transfer Agreement entered into by the Tree of Life collaborator, Genome Research Limited (operating as the Wellcome Sanger Institute) and in some circumstances other Tree of Life collaborators.

## Data Availability

European Nucleotide Archive: Sepioteuthis lessoniana (bigfin reef squid). Accession number PRJEB64979; https://identifiers.org/ena.embl/PRJEB64979. The genome sequence is released openly for reuse. The *Sepioteuthis lessoniana* genome sequencing initiative is part of the Aquatic Symbiosis Genomics (ASG) project ( https://www.ebi.ac.uk/ena/browser/view/PRJEB43743). All raw sequence data and the assembly have been deposited in INSDC databases. Raw data and assembly accession identifiers are reported in Table 1 and Table 2.

## Author information

Members of the Wellcome Sanger Institute Tree of Life Management, Samples and Laboratory Team are listed here: https://doi.org/10.5281/zenodo.13960213.

Members of the Wellcome Sanger Institute Scientific Operations: Sequencing Operations are listed here: https://doi.org/10.5281/zenodo.14870789.

Members of the Wellcome Sanger Institute Tree of Life Core Informatics team are listed here: https://doi.org/10.5281/zenodo.15495178.

Members of the European Bioinformatics Institute ASG Data Portal team are listed here: https://doi.org/10.5281/zenodo.10076466.

Members of the Wellcome Sanger Institute/Aquatic Symbiosis Genomics Project Leadership are listed here: https://doi.org/10.5281/zenodo.10184833.

## References

[ref-1] Abdennur N Mirny LA : Cooler: scalable storage for Hi-C data and other genomically labeled arrays. *Bioinformatics.* 2020;36(1):311–316. 10.1093/bioinformatics/btz540 31290943 PMC8205516

[ref-2] Aken BL Ayling S Barrell D : The ensembl gene annotation system. *Database (Oxford).* 2016;2016: baw093. 10.1093/database/baw093 27337980 PMC4919035

[ref-3] Allio R Schomaker-Bastos A Romiguier J : MitoFinder: efficient automated large-scale extraction of mitogenomic data in target enrichment phylogenomics. *Mol Ecol Resour.* 2020;20(4):892–905. 10.1111/1755-0998.13160 32243090 PMC7497042

[ref-4] Altschul SF Gish W Miller W : Basic Local Alignment Search Tool. *J Mol Biol.* 1990;215(3):403–410. 10.1016/S0022-2836(05)80360-2 2231712

[ref-5] Arnold JM : Embryonic development of the squid.In: Gilbert, D. L., Adelman, W. J., and Arnold, J. M. (eds.) *Squid as experimental animals.*Boston, MA: Springer US,1990;77–90. 10.1007/978-1-4899-2489-6_6

[ref-6] Bateman A Martin MJ Orchard S : UniProt: the Universal Protein Knowledgebase in 2023. *Nucleic Acids Res.* 2023;51(D1):D523–D531. 10.1093/nar/gkac1052 36408920 PMC9825514

[ref-7] Bates A Clayton-Lucey I Howard C : Sanger Tree of Life HMW DNA fragmentation: diagenode Megaruptor ^®^3 for LI PacBio. *protocols.io.* 2023. 10.17504/protocols.io.81wgbxzq3lpk/v1

[ref-8] Benoit G Raguideau S James R : High-quality metagenome assembly from long accurate reads with metaMDBG. *Nat Biotechnol.* 2024;42(9):1378–1383. 10.1038/s41587-023-01983-6 38168989 PMC11392814

[ref-9] Buchfink B Reuter K Drost HG : Sensitive protein alignments at Tree-of-Life scale using DIAMOND. *Nat Methods.* 2021;18(4):366–368. 10.1038/s41592-021-01101-x 33828273 PMC8026399

[ref-10] Challis R Kumar S Sotero-Caio C : Genomes on a Tree (GoaT): a versatile, scalable search engine for genomic and sequencing project metadata across the eukaryotic Tree of Life [version 1; peer review: 2 approved]. *Wellcome Open Res.* 2023;8:24. 10.12688/wellcomeopenres.18658.1 36864925 PMC9971660

[ref-11] Challis R Richards E Rajan J : BlobToolKit – interactive quality assessment of genome assemblies. *G3 (Bethesda).* 2020;10(4):1361–1374. 10.1534/g3.119.400908 32071071 PMC7144090

[ref-12] Chaumeil PA Mussig AJ Hugenholtz P : GTDB-Tk v2: memory friendly classification with the genome taxonomy database. *Bioinformatics.* 2022;38(23):5315–5316. 10.1093/bioinformatics/btac672 36218463 PMC9710552

[ref-13] Cheng H Concepcion GT Feng X : Haplotype-resolved *de novo* assembly using phased assembly graphs with hifiasm. *Nat Methods.* 2021;18(2):170–175. 10.1038/s41592-020-01056-5 33526886 PMC7961889

[ref-14] Cheng SH Anderson FE Bergman A : Molecular evidence for co-occurring cryptic lineages within the *Sepioteuthis cf*. *lessoniana* species complex in the Indian and Indo-West Pacific Oceans. *Hydrobiologia.* 2014;725(1):165–188. 10.1007/s10750-013-1778-0

[ref-15] da Veiga Leprevost F Grüning BA Alves Aflitos S : BioContainers: an open-source and community-driven framework for software standardization. *Bioinformatics.* 2017;33(16):2580–2582. 10.1093/bioinformatics/btx192 28379341 PMC5870671

[ref-16] Danecek P Bonfield JK Liddle J : Twelve years of SAMtools and BCFtools. *GigaScience.* 2021;10(2): giab008. 10.1093/gigascience/giab008 33590861 PMC7931819

[ref-17] DeMaere MZ Darling AE : bin3C: exploiting Hi-C sequencing data to accurately resolve metagenome-assembled genomes. *Genome Biol.* 2019;20(1): 46. 10.1186/s13059-019-1643-1 30808380 PMC6391755

[ref-18] Denton A Yatsenko H Jay J : Sanger Tree of Life wet laboratory protocol collection V.1. *protocols.io.* 2023. 10.17504/protocols.io.8epv5xxy6g1b/v1

[ref-19] Diesh C Stevens GJ Xie P : JBrowse 2: a modular genome browser with views of synteny and structural variation. *Genome Biol.* 2023;24(1): 74. 10.1186/s13059-023-02914-z 37069644 PMC10108523

[ref-20] do Amaral RJV Bates A Denton A : Sanger Tree of Life RNA extraction: automated MagMax ^TM^ mirVana. *protocols.io.* 2023. 10.17504/protocols.io.6qpvr36n3vmk/v1

[ref-21] Ewels P Magnusson M Lundin S : MultiQC: summarize analysis results for multiple tools and samples in a single report. *Bioinformatics.* 2016;32(19):3047–3048. 10.1093/bioinformatics/btw354 27312411 PMC5039924

[ref-22] Ewels PA Peltzer A Fillinger S : The nf-core framework for community-curated bioinformatics pipelines. *Nat Biotechnol.* 2020;38(3):276–278. 10.1038/s41587-020-0439-x 32055031

[ref-23] Formenti G Abueg L Brajuka A : Gfastats: conversion, evaluation and manipulation of genome sequences using assembly graphs. *Bioinformatics.* 2022;38(17):4214–4216. 10.1093/bioinformatics/btac460 35799367 PMC9438950

[ref-24] Grüning B Dale R Sjödin A : Bioconda: sustainable and comprehensive software distribution for the life sciences. *Nat Methods.* 2018;15(7):475–476. 10.1038/s41592-018-0046-7 29967506 PMC11070151

[ref-25] Guan D McCarthy SA Wood J : Identifying and removing haplotypic duplication in primary genome assemblies. *Bioinformatics.* 2020;36(9):2896–2898. 10.1093/bioinformatics/btaa025 31971576 PMC7203741

[ref-26] Harry E : PretextView (Paired REad TEXTure Viewer): a desktop application for viewing pretext contact maps.2022. Reference Source

[ref-27] Howe K Chow W Collins J : Significantly improving the quality of genome assemblies through curation. *GigaScience.* 2021;10(1): giaa153. 10.1093/gigascience/giaa153 33420778 PMC7794651

[ref-28] Imai H Aoki M : Genetic diversity and genetic heterogeneity of bigfin reef squid “ *Sepioteuthis lessoniana*” species complex in Northwestern Pacific Ocean.In: Caliskan, M. (ed.) *Analysis of genetic variation in animal.*InTech,2012. 10.5772/35024

[ref-29] Jay J Yatsenko H Narváez-Gómez JP : Sanger Tree of Life sample preparation: triage and dissection. *protocols.io.* 2023. 10.17504/protocols.io.x54v9prmqg3e/v1

[ref-30] Kang DD Li F Kirton E : MetaBAT 2: an adaptive binning algorithm for robust and efficient genome reconstruction from metagenome assemblies. *PeerJ.* 2019;7:e7359. 10.7717/peerj.7359 31388474 PMC6662567

[ref-31] Kerpedjiev P Abdennur N Lekschas F : HiGlass: web-based visual exploration and analysis of genome interaction maps. *Genome Biol.* 2018;19(1): 125. 10.1186/s13059-018-1486-1 30143029 PMC6109259

[ref-32] Kurtzer GM Sochat V Bauer MW : Singularity: scientific containers for mobility of compute. *PLoS One.* 2017;12(5): e0177459. 10.1371/journal.pone.0177459 28494014 PMC5426675

[ref-33] Letunic I Bork P : Interactive Tree of Life (iTOL) v6: recent updates to the phylogenetic tree display and annotation tool. *Nucleic Acids Res.* 2024;52(W1):W78–W82. 10.1093/nar/gkae268 38613393 PMC11223838

[ref-34] Li H : Minimap2: pairwise alignment for nucleotide sequences. *Bioinformatics.* 2018;34(18):3094–3100. 10.1093/bioinformatics/bty191 29750242 PMC6137996

[ref-35] Manni M Berkeley MR Seppey M : BUSCO update: novel and streamlined workflows along with broader and deeper phylogenetic coverage for scoring of eukaryotic, prokaryotic, and viral genomes. *Mol Biol Evol.* 2021;38(10):4647–4654. 10.1093/molbev/msab199 34320186 PMC8476166

[ref-36] Merkel D : Docker: lightweight Linux containers for consistent development and deployment. *Linux J.* 2014;2014(239):2, [Accessed 2 April 2024]. Reference Source

[ref-37] Nakajima R Lajbner Z Kuba MJ : Squid adjust their body color according to substrate. *Sci Rep.* 2022;12(1): 5227. 10.1038/s41598-022-09209-6 35347207 PMC8960755

[ref-38] Narváez-Gómez JP Mbye H Oatley G : Sanger Tree of Life sample homogenisation: covaris cryoPREP ^®^ automated dry pulverizer V.1. *protocols.io.* 2023. 10.17504/protocols.io.eq2lyjp5qlx9/v1

[ref-39] Oatley G Denton A Howard C : Sanger Tree of Life HMW DNA extraction: automated MagAttract v.2. *protocols.io.* 2023. 10.17504/protocols.io.kxygx3y4dg8j/v1

[ref-40] Olm MR Brown CT Brooks B : dRep: a tool for fast and accurate genomic comparisons that enables improved genome recovery from metagenomes through de-replication. *ISME J.* 2017;11(12):2864–2868. 10.1038/ismej.2017.126 28742071 PMC5702732

[ref-41] Parks DH Imelfort M Skennerton CT : CheckM: assessing the quality of microbial genomes recovered from isolates, single cells, and metagenomes. *Genome Res.* 2015;25(7):1043–55. 10.1101/gr.186072.114 25977477 PMC4484387

[ref-42] Pointon DL Eagles W Sims Y : sanger-tol/treeval v1.0.0 – Ancient Atlantis.2023. 10.5281/zenodo.10047654

[ref-43] Quinlan AR Hall IM : BEDTools: a flexible suite of utilities for comparing genomic features. *Bioinformatics.* 2010;26(6):841–842. 10.1093/bioinformatics/btq033 20110278 PMC2832824

[ref-44] Ranallo-Benavidez TR Jaron KS Schatz MC : GenomeScope 2.0 and Smudgeplot for reference-free profiling of polyploid genomes. *Nat Commun.* 2020;11(1): 1432. 10.1038/s41467-020-14998-3 32188846 PMC7080791

[ref-45] Rao SSP Huntley MH Durand NC : A 3D map of the human genome at kilobase resolution reveals principles of chromatin looping. *Cell.* 2014;159(7):1665–1680. 10.1016/j.cell.2014.11.021 25497547 PMC5635824

[ref-46] Rhie A Walenz BP Koren S : Merqury: reference-free quality, completeness, and phasing assessment for genome assemblies. *Genome Biol.* 2020;21(1): 245. 10.1186/s13059-020-02134-9 32928274 PMC7488777

[ref-47] Rühlemann MC Wacker EM Ellinghaus D : MAGScoT: a fast, lightweight and accurate bin-refinement tool. *Bioinformatics.* 2022;38(24):5430–5433. 10.1093/bioinformatics/btac694 36264141 PMC9750101

[ref-48] Sakurai Y Ikeda Y : Effect of visual lateralization on the spatial position of individuals within a school of oval squid ( *Sepioteuthis lessoniana*). *J Comp Physiol A Neuroethol Sens Neural Behav Physiol.* 2024;210(3):381–398. 10.1007/s00359-023-01654-6 37515730

[ref-49] Seemann T : Prokka: rapid prokaryotic genome annotation. *Bioinformatics.* 2014;30(14):2068–2069. 10.1093/bioinformatics/btu153 24642063

[ref-50] Shigeno S Tsuchiya K Segawa S : Conserved topological patterns and heterochronies in loliginid cephalopods: comparative developmental morphology of the oval squid *Sepioteuthis lessoniana*. *Invertebr Reprod Dev.* 2001;39(3):161–174. 10.1080/07924259.2001.9652481

[ref-51] Strickland M Cornwell C Howard C : Sanger Tree of Life fragmented DNA clean up: manual SPRI. *protocols.io.* 2023. 10.17504/protocols.io.kxygx3y1dg8j/v1

[ref-52] Sugimoto C Ikeda Y : Ontogeny of schooling behavior in the oval squid *Sepioteuthis lessoniana*. * Fish Sci.* 2012;78(2):287–294. 10.1007/s12562-011-0464-2

[ref-53] Sugimoto C Yanagisawa R Nakajima R : Observations of schooling behaviour in the oval squid *Sepioteuthis lessoniana* in coastal waters of Okinawa Island. *Mar Biodivers Rec.* 2013;6:e34. 10.1017/S1755267213000067

[ref-54] Tomano S Sanchez G Kawai K : Contribution of *Sepioteuthis* sp. 1 and *Sepioteuthis* sp. 2 to oval squid fishery stocks in western Japan. *Fish Sci.* 2016;82(4):585–596. 10.1007/s12562-016-0988-6

[ref-55] Uliano-Silva M Ferreira JGRN Krasheninnikova K : MitoHiFi: a python pipeline for mitochondrial genome assembly from PacBio high fidelity reads. *BMC Bioinformatics.* 2023;24(1): 288. 10.1186/s12859-023-05385-y 37464285 PMC10354987

[ref-56] UniProt Consortium: UniProt: a worldwide hub of protein knowledge. *Nucleic Acids Res.* 2019;47(D1):D506–D515. 10.1093/nar/gky1049 30395287 PMC6323992

[ref-57] Vasimuddin M Misra S Li H : Efficient architecture-aware acceleration of BWA-MEM for multicore systems.In: *2019 IEEE International Parallel and Distributed Processing Symposium (IPDPS).*IEEE,2019;314–324. 10.1109/IPDPS.2019.00041

[ref-58] Wu YW Tang YH Tringe SG : MaxBin: an automated binning method to recover individual genomes from metagenomes using an expectation-maximization algorithm. *Microbiome.* 2014;2(1): 26. 10.1186/2049-2618-2-26 25136443 PMC4129434

[ref-59] Zhou C McCarthy SA Durbin R : YaHS: yet another Hi-C scaffolding tool. *Bioinformatics.* 2023;39(1): btac808. 10.1093/bioinformatics/btac808 36525368 PMC9848053

