# Supplementary material for: The chromosomal genome sequence of the bigfin reef squid, Sepioteuthis lessoniana d'Orbigny, 1826 and its associated microbial metagenome sequences
Source: Wellcome Open Res. 2025 Jul 14;10:351. [Version 1] doi: 10.12688/wellcomeopenres.24553.1 (PMC12780523; doi:10.12688/wellcomeopenres.24553.1)
Supplement: Peer review history

# Reviewer report: Reviewer response for version 1

**DOI:** 10.21956/wellcomeopenres.27060.r142580

**Published:** 2026-01-09

**Authors:** Ole Kristian Tørresen

**Affiliations:** 1 Department of Biosciences, University of Oslo CEES (Ringgold ID: 206081), Oslo, Oslo, Norway

The authors have sequenced and assembled the genome of bigfin reef squid, a squid occurring in the Indo-Pacific Ocean. 

 The data note follows the usual setup, and is in general concise and to the point for most information, but with some details lacking. The biggest issue I have with this data note is the metagenomic part. 

 What is the reason for doing this particular analysis? Is it known that these squids have lots of different bacteria in their tissues? (Which seems quite strange.)  

 On which sample (tissue) was this performed? This is not stated as far as I can see. Both xcSepLess1 and xcSepLess2 are from somatic tissue, and as far as I understand, should not contain a lot of bacteria.

 As it is now, the metagenomic part of the data note seems disconnected from the rest, with no justification and lacking in detail around the source of the sample.

Are sufficient details of methods and materials provided to allow replication by others?

Yes

Is the rationale for creating the dataset(s) clearly described?

Partly

Are the datasets clearly presented in a useable and accessible format?

Yes

Are the protocols appropriate and is the work technically sound?

Yes

Reviewer Expertise:

Genome assembly

I confirm that I have read this submission and believe that I have an appropriate level of expertise to confirm that it is of an acceptable scientific standard, however I have significant reservations, as outlined above.

# Reviewer report: Reviewer response for version 1

**DOI:** 10.21956/wellcomeopenres.27060.r142577

**Published:** 2026-01-07

**Authors:** David EK Ferrier

**Affiliations:** 1 University of St Andrews, Scotland, UK

The rationale is clear.  Besides the need for sequencing this mollusc as part of the Earth Biogenome Project and the Aquatic Symbiosis Initiative, there is the added interest of this animal being amenable to lab culture and hence experimental approaches for a variety of biological questions as well as aquaculture (as summarized briefly in the Introduction).

 The various protocols are certainly appropriate and the work is of a high technical standard.

 The materials and methods descriptions meet accepted standards for these data notes, and the datasets such as the assemblies of the two haplotypes are accessible and useable.

 There are a couple of minor typos for correction.

 1) Typo in Background, third line: insert ‘to’ to read, “that belongs to the”.

 2) Genome sequence report section, first paragraph, line 8: delete an unnecessary hyphen to read, “generate”.

Are sufficient details of methods and materials provided to allow replication by others?

Yes

Is the rationale for creating the dataset(s) clearly described?

Yes

Are the datasets clearly presented in a useable and accessible format?

Yes

Are the protocols appropriate and is the work technically sound?

Yes

Reviewer Expertise:

NA

I confirm that I have read this submission and believe that I have an appropriate level of expertise to confirm that it is of an acceptable scientific standard.

# Reviewer report: Reviewer response for version 1

**DOI:** 10.21956/wellcomeopenres.27060.r142579

**Published:** 2026-01-02

**Authors:** Erich M. Schwarz

**Affiliations:** 1 Cornell University, Ithaca, New York, USA

The genome assemblies of Sanchez et al. are well done and well described.  I have only a few questions.

 1. What is the correct full name for the species being analyzed?  In the title, the authors refer to it as " *Sepioteuthis lessoniana *d'Orbigny, 1826"; but at the start of the Background, the authors refer to it as " *Sepioteuthis lessoniana Férussac*, 1831".  Which of these full species names is correct, and could the authors please stick to the correct one?

 2. In the Sample Acquisition section of the Methods, the authors give a species identification for the specimen used in genome sequencing ("one female of Sepioteuthis sp. 2"), but do not give a species identification for the specimen used in Hi-C and RNA-sequencing ("an adult specimen").  Could the authors provide a species identification for the second specimen?

 3. Would it be possible to provide a scale bar for Figure 1?

Are sufficient details of methods and materials provided to allow replication by others?

Yes

Is the rationale for creating the dataset(s) clearly described?

Yes

Are the datasets clearly presented in a useable and accessible format?

Yes

Are the protocols appropriate and is the work technically sound?

Yes

Reviewer Expertise:

Nematode genome assembly and analysis

I confirm that I have read this submission and believe that I have an appropriate level of expertise to confirm that it is of an acceptable scientific standard.
